# Supplementary material for: Antibacterial Properties of Peptide and Protein Fractions from Cornu aspersum Mucus
Source: Molecules. 2024 Jun 18;29(12):2886. doi: 10.3390/molecules29122886 (PMC11206429; doi:10.3390/molecules29122886)
Supplement: Supplementary file 1 [file molecules-29-02886-s001.zip › molecules-3007928-supplementary.pdf]

Supplementary Information:

SI S1. An alignment of amino acid sequences of some identified peptides from *C. aspersum* mucus with known antimicrobial peptides with data base AMPs by CAMPSing, Matrix: BLOSUM 80 (http://www.campsign.bicnirrh.res.in/blast.php, accessed on 24 March 2024) and with proteins by BLAST (https://blast.ncbi.nlm.nih.gov , **a**ccessed on accessed on 24 March 2024).

PEPTIDE 1. LLPFKEPDL

```
> DBAASP_4424|4774|Indolicidin
Length=13, Score = 18.5 bits (34), Expect = 2.4
Identities = 6/9 (67%), Positives = 7/9 (78%), Gaps = 0/9 (0%)

Query  1  ILPFKEPDL  9
          ILP K+P L
Sbjct  1  ILPWKKPKL  9
```

PEPTIDE 3. LNLGGNGANGLVGG

>UniProtKb_253|Q8K4I4|BPIA1_RAT; **BPI fold-containing family A member 1**; Lipid-binding protein which plays a role in the innate immune responses of the upper airways.

```
Length=270, Score = 22.4 bits (43), Expect = 0.66
Identities = 8/11 (73%), Positives = 9/11 (82%), Gaps = 0/11 (0%)

Query  4    GGNGANGLVGG  14
            GG  +NGLVGG
Sbjct  97   GGGSSNGLVGG  107
```

>UniProtKb_251|P97361;Q7TQJ2|BPIA1_MOUSE; **BPI fold-containing family A member 1**; Lipid-binding protein which plays a role in the innate immune responses of the upper airways.

```
Length=278, Score = 22.4 bits (43), Expect = 0.67
Identities = 8/11 (73%), Positives = 9/11 (82%), Gaps = 0/11 (0%)

Query  4    GGNGANGLVGG  14
            GG  +NGLVGG
Sbjct  105  GGGNSNGLVGG  115
```

PEPTIDE 4. AGVGGAAGNPSTYVG

>CAMPSQ2894; **Enterocin E-760**; **AMP Family: Bacteriocin**; Source: Bacteria *Enterococcus durans*; Activity: Gram+ve, Gram-ve

```
Length=62, Score = 20.2 bits (38), Expect = 2.5
Identities = 7/7 (100%), Positives = 7/7 (100%), Gaps = 0/7 (0%)

Query  2   GVGGAAG  8
           GVGGAAG
Sbjct  11  GVGGAAG  17
```

PEPTIDE 6. MLGGGVNSLRPPK

>CAMPSQ2582; **Hyastatin**; Source: *Hyas araneus* [Great spider crab] Activity: Antibacterial, Antifungal; Gram+ve, Gram-ve

```
Length=131, Score = 21.5 bits (41), Expect = 1.1
Identities = 7/10 (70%), Positives = 8/10 (80%), Gaps = 0/10 (0%)

Query  2   LGGGVNSLRP  11
           LGGG+ S RP
Sbjct  79  LGGGIGSTRP  88
```

PEPTIDE 7. CVGGAGGHGDSCAKGT

>APD_2350|AP02350|**Microcin B;** AMP Family: Bacteriocin; Activity: Antimicrobial

Length=42, Score = 20.6 bits (39), Expect = 1.6
Identities = 7/10 (70%), Positives = 7/10 (70%), Gaps = 0/10 (0%)

```
Query  3   GGAGGHGDSC  12
           GG GG G SC
Sbjct  6   GGGGGGGGSC  15
```

> CAMPSQ1503 **Bacteriocin microcin B17**; AMP Family: Bacteriocin; Source: *Escherichia coli*; Activity: Antimicrobial
Length=43, Score = 20.6 bits (39), Expect = 1.6
Identities = 7/10 (70%), Positives = 7/10 (70%), Gaps = 0/10 (0%)

```
Query  3   GGAGGHGDSC  12
           GG GG G SC
Sbjct  6   GGGGGGGGSC  15
```

> LAMP_Experimental_1801|L06AT00084|Q5Z434_ORYSJ
Length=45, Score = 20.6 bits (39), Expect = 1.7
Identities = 7/10 (70%), Positives = 8/10 (80%), Gaps = 0/10 (0%)

```
Query  5   AGGHGDSCAK  14
           AGG  D+CAK
Sbjct  19  AGGSRDTCAK  28
```

PEPTIDE 8. GGGGYHTWGEGGKF

> UniProtKb_1144|Q8RYE7;Q6ZZS3|DF196_ARATH: **Defensin-like protein 196;** Organism *Arabidopsis thaliana*
Length=97, Score = 22.4 bits (43), Expect = 0.53
Identities = 8/11 (73%), Positives = 8/11 (73%), Gaps = 0/11 (0%)

```
Query  3   GGYHTWGEGGK  13
           GG   WGEGGK
Sbjct  67  GGKCLWGEGGK  77
```

> Yadamp_463|1414|**Glycine-rich protein GWK isoform 3**
Length=34, Score = 20.6 bits (39), Expect = 1.1
Identities = 8/13 (62%), Positives = 9/13 (69%), Gaps = 0/13 (0%)

```
Query  1   GGGGYHTWGEGGK  13
           GGGG+   G GGK
Sbjct  20  GGGGWKGGGGGGK  32
```

> Yadamp_508|1455|**Glycine-rich protein GWK isoform 2**
Length=35, Score = 20.6 bits (39), Expect = 1.1
Identities = 8/13 (62%), Positives = 9/13 (69%), Gaps = 0/13 (0%)

```
Query  1   GGGGYHTWGEGGK  13
           GGGG+   G GGK
Sbjct  20  GGGGWKGGGGGGK  32
```

> CAMPSQ3973 **Glycine-rich protein GWK;** Source: *Cucumis melo* [Muskmelon]; Activity: Antifungal
Length=36, Score = 20.6 bits (39), Expect = 1.1
Identities = 8/13 (62%), Positives = 9/13 (69%), Gaps = 0/13 (0%)

```
Query  1   GGGGYHTWGEGGK  13
           GGGG+   G GGK
Sbjct  20  GGGGWKGGGGGGK  32
```

PEPTIDE 10. NLVGGSGGGGRGGANPLG

> APD_1398|AP02256|**Procambarin,** Activity: Antibacterial

Length=155, Score = 28.4 bits (57), Expect = 0.008
Identities = 10/15 (67%), Positives = 11/15 (73%), Gaps = 0/15 (0%)

```
Query  4   GGSGGGGRGGANPLG  18
           GG GGGG GG  P+G
Sbjct  26  GGFGGGGLGGGKPIG  40
```

> CAMPSQ322; **Holotricin-3**; Source: *Holotrichia diomphalia* Activity: Antibacterial, Antifungal
Length=84; Score = 26.7 bits (53), Expect = 0.022
Identities=9/10 (90%), Positives=9/10 (90%), Gaps=0/10 (0%)

```
Query  4   GGSGGGGRGG  13
           GG GGGGRGG
Sbjct  41  GGHGGGGRGG  50
```

> APD_2350|AP02350|Microcin B; AMP Family: Bacteriocin; Activity: Antimicrobial
Length=42; Score = 24.5 bits (48), Expect = 0.060
Identities = 8/12 (67%), Positives = 10/12 (83%), Gaps = 0/12 (0%)

```
Query  3   VGGSGGGGRGGA  14
           +GG GGGG GG+
Sbjct  3   IGGGGGGGGGGS  14
```

PEPTIDE 11. GTMSPAGGEMGPVTAGVG

> UniProtKb_2464|A1XQR6|ROMO1_PIG; Reactive oxygen species modulator 1; It has antibacterial activity against a variety of bacteria including *S.aureus*, P.*aeruginosa* and *M.tuberculosis*. Acts by inducing bacterial membrane breakage (By similarity).
Length=79, Score = 20.2 bits (38), Expect = 3.2
Identities = 9/18 (50%), Positives = 11/18 (61%), Gaps = 1/18 (6%)

```
Query  2   TMSPAGGEMGPVTA-GVG  18
           TM  +GG  GP  A G+G
Sbjct  59  TMMQSGGTFGPFMAIGMG  76
```

PEPTIDE 12. GTKGCGPGSCPPGDTVAGVG

> CAMPSQ2192; **Crustin-like antimicrobial peptide**; Source: *Fenneropenaeus indicus* Activity: Antimicrobial
Length=117, Score = 25.0 bits (49), Expect = 0.11
Identities = 9/16 (56%), Positives = 10/16 (63%), Gaps = 1/16 (6%)

```
Query  5   CGPGSCPP-GDTVAGV  19
           C PG CPP  DT  G+
Sbjct  63  CKPGRCPPVRDTCPGI  78
```

> CAMPSQ3075 **Gallinacin 8**; Source: *Gallus gallus [Chicken]*; Activity: Antimicrobial
Length=41, Score = 23.7 bits (46), Expect = 0.14
Identities = 7/14 (50%), Positives = 10/14 (71%), Gaps = 0/14 (0%)

```
Query  5   CGPGSCPPGDTVAG  18
           C  G+CPP  T++G
Sbjct  12  CRAGACPPTFTISG  25
```

> APD_166|AP01147|**Gallinacin 8,** Activity: Antimicrobial
Length=41, Score = 23.7 bits (46), Expect = 0.14
Identities = 7/14 (50%), Positives = 10/14 (71%), Gaps = 0/14 (0%)

```
Query  5   CGPGSCPPGDTVAG  18
           C  G+CPP  T++G
Sbjct  12  CRAGACPPTFTISG  25
```

> CAMPSQ1841 Gallinacin-10; Source: *Gallus gallus*; Activity: Antibacterial
Length=43, Score = 23.7 bits (46), Expect = 0.15
Identities = 7/14 (50%), Positives = 10/14 (71%), Gaps = 0/14 (0%)

```
Query  5   CGPGSCPPGDTVAG  18
           C  G+CPP  T++G
Sbjct  14  CRAGACPPTFTISG  27
```

> UniProtKb_877|Q2V433|DEF52_ARATH; **Putative defensin-like protein 52; Arabidopsis thaliana (Mouse-ear cress)**
Length=81, Score = 24.1 bits (47), Expect = 0.18
Identities = 10/19 (53%), Positives = 11/19 (58%), Gaps = 0/19 (0%)

```
Query  2   TKGCGPGSCPPGDTVAGVG  20
           T+G   GSC PG T A  G
Sbjct  47  TEGYDDGSCVPGPTTAKCG  65
```

> UniProtKb_1513|Q6QLQ9|GLL10_CHICK; Gallinacin 10
Length=68, Score = 23.7 bits (46), Expect = 0.23
Identities = 7/14 (50%), Positives = 10/14 (71%), Gaps = 0/14 (0%)

```
Query  5   CGPGSCPPGDTVAG  18
           C  G+CPP  T++G
Sbjct  39  CRAGACPPTFTISG  52
```

> CAMPSQ1080 Gallinacin-4; Source: *Gallus gallus*; Activity: Antibacterial
Length=38, Score = 22.8 bits (44), Expect = 0.28
Identities = 7/15 (47%), Positives = 9/15 (60%), Gaps = 0/15 (0%)

```
Query  5   CGPGSCPPGDTVAGV  19
           C PG CP G+   G+
Sbjct  13  CTPGKCPHGNAYLGL  27
```

> CAMPSQ2874 Chicken AvBD4; Activity: Antibacterial; Gram-ve
Length=38, Score = 22.4 bits (43), Expect = 0.42
Identities = 7/15 (47%), Positives = 9/15 (60%), Gaps = 0/15 (0%)

```
Query  5   CGPGSCPPGDTVAGV  19
           C PG CP G+   G+
Sbjct  13  CTPGKCPYGNAYLGL  27
```

> CAMPSQ4487 Basic endochitinase B; Activity: Antifungal
Length=255, Score = 22.8 bits (44), Expect = 0.62
Identities = 7/11 (64%), Positives = 7/11 (64%), Gaps = 0/11 (0%)

```
Query  5   CGPGSCPPGDT  15
           C PG  PPG T
Sbjct  40  CTPGGTPPGPT  50
```

> UniProtKb_483|P19171;Q9S7J5;Q9S838;Q9SXJ2;Q9SXJ3;Q9SXJ4|CHIB_ARATH
Length=335, Score = 22.8 bits (44), Expect = 0.63
Identities = 7/11 (64%), Positives = 7/11 (64%), Gaps = 0/11 (0%)

```
Query  5   CGPGSCPPGDT  15
           C PG  PPG T
Sbjct  73  CTPGGTPPGPT  83
```

PEPTIDE 13. ACSLLLGGGGVGGGKGGGGHAG

> CAMPSQ3913, Ctenidin-1 from *Cupiennius salei* [Wandering spider], Activity: Antimicrobial
Length=119, Score = 37.0 bits (77), Expect = 9e-006, Identities = 12/16 (75%), Positives = 15/16 (94%), Gaps = 0/16 (0%)

```
P 9    7    GGGGVGGGKGGGGHAG  22
            GGGG+GGG+GGGG+ G
Sbjct  88   GGGGLGGGRGGGGYGG  103
```

> CAMPSQ3914, Ctenidin-3 from *Cupiennius salei* [Wandering spider], Activity: Antimicrobial
Length=120,Score = 37.0 bits (77),Expect = 1e-005,Identities = 12/16 (75%), Positives = 15/16 (94%), Gaps = 0/16 (0%)

```
P 9    7    GGGGVGGGKGGGGHAG  22
            GGGG+GGG+GGGG+ G
Sbjct  90   GGGGLGGGRGGGGYGG  105
```

> DBAASP_2873|3113|Acanthoscurrin-2 from *Acanthoscurria gomesiana* [Tarantula spider], Activity: Antibacterial (Gram-), Antifungal, Target: *E.coli* SBS363, *C.albicans*
Length=130, Score = 33.1 bits (68), Expect = 2e-004, Identities = 13/20 (65%), Positives = 16/20 (80%), Gaps = 3/20 (15%)

```
P 9    6    LGGGGVGGGK---GGGGHAG  22
            LGGGG+GGG+   GGGG+ G
Sbjct  99   LGGGGLGGGRGGYGGGGYGG  118
```

> CAMPSQ1508, Acanthoscurrin-1 from *Acanthoscurria gomesiana* [Tarantula spider], Activity: Antibacterial (Gram-), Antifungal, Target: *E.coli* SBS363, *C.albicans*
Length=132, Score = 32.7 bits (67), Expect = 3e-004, Identities = 14/19 (74%), Positives = 15/19 (79%), Gaps = 2/19 (11%)

```
P 9    6   LGGGGVGGGK--GGGGHAG  22
           LGGGG+GGGK  GGGG  G
Sbjct  31  LGGGGLGGGKGLGGGGLGG  49
```

PEPTIDE 14. LLLDGFGGGLLVEHDPGS

> CAMPSQ3509 Prolixicin Source: *Rhodnius prolixus* [Triatomid bug]; Activity: Antibacterial; Gram+ve, Gram-ve
Length=77, Score = 19.8 bits (37), Expect = 4.5
Identities = 5/9 (56%), Positives = 8/9 (89%), Gaps = 0/9 (0%)

```
Query  6   FGGGLLVEH  14
           +GGG+ +EH
Sbjct  65  YGGGMRIEH  73
```

> APD_888|AP01798|Prolixicin
Length=77, Score = 19.8 bits (37), Expect = 4.5
Identities = 5/9 (56%), Positives = 8/9 (89%), Gaps = 0/9 (0%)

```
Query  6   FGGGLLVEH  14
           +GGG+ +EH
Sbjct  65  YGGGMRIEH  73
```

PEPTIDE 15. MGGWGGLGGGHNGGWMPPK

> CAMPSQ959; Shepherin II; Source: *Capsella bursa-pastoris* [Shepherd purse]; Activity: Antibacterial, Antifungal; Gram+ve, Gram-ve
Length=38, Score = 28.0 bits (56), Expect = 0.003
Identities = 10/13 (77%), Positives = 11/13 (85%), Gaps = 0/13 (0%)

```
Query  2   GGWGGLGGGHNGG  14
           GG GG GGG+NGG
Sbjct  4   GGHGGHGGGYNGG  16
```

> APD_1790|AP00512|Shepherin II
Length=38, Score = 28.0 bits (56), Expect = 0.003
Identities = 10/13 (77%), Positives = 11/13 (85%), Gaps = 0/13 (0%)

```
Query  2   GGWGGLGGGHNGG  14
           GG GG GGG+NGG
Sbjct  4   GGHGGHGGGYNGG  16
```

> AMSDb_802|SHEP_CAPBU|ANTIMICROBIAL PEPTIDE SHEP-GRP. SHEPHERIN-1;
Length=120 Score = 28.0 bits (56), Expect = 0.010
Identities = 10/13 (77%), Positives = 11/13 (85%), Gaps = 0/13 (0%)

```
Query  2   GGWGGLGGGHNGG  14
           GG GG GGG+NGG
Sbjct  75  GGHGGHGGGYNGG  87
```

PEPTIDE 16. ACLTPVDHFFAGMPCGGGP

> CAMPSQ5060, **Cyclotide 2a** from *Viola baoshanensis,* Activity: Antimicrobial
Length=113, Score = 20.2 bits (38), Expect = 3.6,Identities = 8/16 (50%), Positives = 10/16 (63%), Gaps = 0/16 (0%)

```
P 10   1   ACLTPVDHFFAGMPCG  16
           A L  +DH   G+PCG
Sbjct  69  ALLKNLDHGRNGVPCG  84
```

PEPTIDE 17. NGLFGGLGGGGHGGGGKGPGEGGG

> CAMPSQ322, Holotricin-3 from *Holotrichia diomphalia* [Korean black chafer], Activity: **Antibacterial, Antifungal**
Length=84, Score = 39.2 bits (82),Expect = 1e-006,Identities = 16/23 (70%), Positives = 17/23 (74%), Gaps = 0/23 (0%)

```
P 11   2   GLFGGLGGGGHGGGGKGPGEGGG  24
           G FGG  GGGHGGGG+G G  GG
Sbjct  33  GGFGGGHGGGHGGGGRGGGGSGG  55
```

> CAMPSQ3914, Ctenidin-3 from *Cupiennius salei* [Wandering spider], Activity: **Antimicrobial**
Length=120, Score = 38.8 bits (81),Expect = 3e-006,Identities = 15/20 (75%), Positives = 16/20 (80%), Gaps = 0/20 (0%)

```
P 11   5   GGLGGGGHGGGGKGPGEGGG  24
           GG GGGG+GGGG G G GGG
Sbjct  11  GGYGGGGYGGGGGGYGGGGG  30
```

> CAMPSQ3913 Ctenidin-1 from *Cupiennius salei* [Wandering spider], Activity: Antimicrobial
Length=119, Score = 37.9 bits (79), Expect = 5e-006,Identities = 15/20 (75%), Positives = 16/20 (80%), Gaps = 0/20 (0%)

```
P 11   5   GGLGGGGHGGGGKGPGEGGG  24
           GGLGGG  GGGG G G+GGG
Sbjct  70  GGLGGGQGGGGGLGGGQGGG  89
```

> CAMPSQ1508, Acanthoscurrin-1 from *Acanthoscurria gomesiana* [Tarantula spider] Activity: Antibacterial (Gram-), Antifungal, Target: *E.coli* SBS363, *C.albicans*
Length=132, Score = 37.5 bits (78), Expect = 8e-006, Identities = 15/19 (79%), Positives = 15/19 (79%), Gaps = 0/19 (0%)

```
P 11   6    GLGGGGHGGGGKGPGEGGG  24
            GLGGGG GGGG G G GGG
Sbjct  93   GLGGGGLGGGGLGGGRGGG  111
```

PEPTIDE 18. LLLDNKGGGLVGGLLGGGGKGGG

> CAMPSQ3894 **Leptoglycin; Source: *Leptodactylus pentadactylus* [Smokey jungle frog] Activity: Antibacterial; Gram-ve**
Length=22, Score = 33.1 bits (68), Expect = 5e-005
Identities = 13/16 (81%), Positives = 14/16 (88%), Gaps = 0/16 (0%)

```
Query  8   GGLVGGLLGGGGKGGG  23
           GGL+G LLGGGG GGG
Sbjct  4   GGLLGPLLGGGGGGGG  19
```

> DBAASP_2873|3113|Acanthoscurrin-2; Source: *Acanthoscurria gomesiana* [Tarantula spider] Activity: Antibacterial, Antifungal; Gram-ve
Length=130, Score = 33.6 bits (69), Expect = 2e-004
Identities = 14/17 (82%), Positives = 14/17 (82%), Gaps = 0/17 (0%)

```
Query  7   GGGLVGGLLGGGGKGGG  23
           GGGL GG LGGGG GGG
Sbjct  23  GGGLGGGGLGGGGLGGG  39
```

> CAMPSQ1508 Acanthoscurrin-1; Source: *Acanthoscurria gomesiana* [Tarantula spider] Activity: Antibacterial, Antifungal; Gram-ve
Length=132 Score = 33.6 bits (69), Expect = 2e-004
Identities = 14/17 (82%), Positives = 14/17 (82%), Gaps = 0/17 (0%)

```
Query  7   GGGLVGGLLGGGGKGGG  23
           GGGL GG LGGGG GGG
Sbjct  23  GGGLGGGGLGGGGLGGG  39
```

PEPTIDE 19. GMVLLHCSPALDFHKTPAV

> CAMPSQ4289; Hepcidin; Source: *Crocodylus siamensis* [Siamese crocodile]; Activity:Antibacterial; Gram+ve, Gram-ve
Length=99, Score = 19.3 bits (36), Expect = 7.0
Identities = 7/9 (78%), Positives = 8/9 (89%), Gaps = 0/9 (0%)

```
Query  2   MVLLHCSPA  10
           +VLLH SPA
Sbjct  13  LVLLHGSPA  21
```

PEPTIDE 20. LPFLLGVGGLLGGSVGGGGGGGGAPL

> CAMPSQ3894 Leptoglycin; Source: *Leptodactylus pentadactylus* [Smokey jungle frog] Activity: Antibacterial; Gram-ve
Length=22, Score = 34.4 bits (71), Expect = 2e-005
Identities = 14/20 (70%), Positives = 16/20 (80%), Gaps = 0/20 (0%)

```
Query  7   VGGLLGGSVGGGGGGGGAPL  26
           +GGLLG  +GGGGGGGG  L
Sbjct  3   LGGLLGPLLGGGGGGGGGLL  22

Sbjct  3   LGGLLGPLLGGGGGGGGGLL  22
```

> DBAASP_2873|3113|Acanthoscurrin-2 Source: *Acanthoscurria gomesiana* [Tarantula spider], Activity: Antibacterial (Gram-), Antifungal, Target: *E.coli* SBS363, *C.albicans*
Length=130, Score = 33.1 bits (68), Expect = 3e-004
Identities = 14/19 (74%), Positives = 15/19 (79%), Gaps = 0/19 (0%)

```
Query  5   LGVGGLLGGSVGGGGGGGG  23
           LG GGL GG +GGGG GGG
Sbjct  42  LGGGGLGGGGLGGGGLGGG  60
```

> CAMPSQ1508 Acanthoscurrin-1 Source: *Acanthoscurria gomesiana* [Tarantula spider], Activity: Antibacterial (Gram-), Antifungal, Target: *E.coli* SBS363, *C.albicans*
Length=132 Score = 33.1 bits (68), Expect = 3e-004
Identities = 14/19 (74%), Positives = 15/19 (79%), Gaps = 0/19 (0%)

```
Query  5   LGVGGLLGGSVGGGGGGGG  23
           LG GGL GG +GGGG GGG
Sbjct  42  LGGGGLGGGGLGGGGLGGG  60
```

PEPTIDE 21. MVLDGKGGGGLLGGVLGGGKDAHLGG

> DBAASP_2873|3113|Acanthoscurrin-2; Source: *Acanthoscurria gomesiana* [Tarantula spider], Activity: Antibacterial (Gram-), Antifungal, Target: E. coli SBS363, C. albicans
Length=130, Score = 32.7 bits (67), Expect = 4e-004
Identities = 14/22 (64%), Positives = 16/22 (73%), Gaps = 0/22 (0%)

```
Query  5    GKGGGGLLGGVLGGGKDAHLGG  26
            G GGGGL GG LGGG+  + GG
Sbjct  93   GLGGGGLGGGGLGGGRGGYGGG  114
```

PEPTIDE 22. LLKDNGVGGLLGGGGAGGGGLVGGNLGGGAG

> DBAASP_2873|3113|Acanthoscurrin-2 (> CAMPSQ1805) Source: *Acanthoscurria gomesiana* [Tarantula spider], Activity: Antibacterial (Gram-), Antifungal, Target: E. coli SBS363, C. albicans
Length=130, Score = 45.2 bits (96), Expect = 2e-008
Identities = 19/24 (79%), Positives = 19/24 (79%), Gaps = 0/24 (0%)

```
Query  8   GGLLGGGGAGGGGLVGGNLGGGAG  31
           GG LGGGG GGGGL GG LGGG G
Sbjct  44  GGGLGGGGLGGGGLGGGGLGGGKG  67
```

> CAMPSQ1508; Acanthoscurrin-1; Source: *Acanthoscurria gomesiana* [Tarantula spider], Activity: Antibacterial (Gram-), Antifungal, Target: E. coli SBS363, C. albicans
Length=132; Score = 31.4 bits (64), Expect = 0.001
Identities = 16/22 (73%), Positives = 16/22 (73%), Gaps = 2/22 (9%)

```
Query  5   GKGGGGLLGGVLGGGKDAHLGG  26
           G GGGGL GG LGGGK   LGG
Sbjct  25  GLGGGGLGGGGLGGGKG--LGG  44
```

PEPTIDE 23. KTSKLMVYLAGGGGGLLGGVGGGGGGAGGGGPGGL

> CAMPSQ3913; Ctenidin-1; Source:*Cupiennius salei* [Wandering spider]; Activity: Antimicrobial
Length=119, Score = 45.7 bits (97), Expect = 2e-008
Identities = 19/24 (79%), Positives = 19/24 (79%), Gaps = 0/24 (0%)

```
Query  11   GGGGGLLGGVGGGGGGAGGGGPGG  34
            GGGGGL GG GGGG G GGGG GG
Sbjct  87   GGGGGLGGGRGGGGYGGGGGGYGG  110
```

> CAMPSQ3914; Ctenidin-3; Source: *Cupiennius salei* [Wandering spider]; Activity: Antimicrobial
Length=120, Score = 45.7 bits (97), Expect = 2e-008
Identities = 19/24 (79%), Positives = 19/24 (79%), Gaps = 0/24 (0%)

```
Query  11   GGGGGLLGGVGGGGGGAGGGGPGG  34
            GGGGGL GG GGGG G GGGG GG
Sbjct  89   GGGGGLGGGRGGGGYGGGGGGYGG  112
```

> Yadamp_463|1414|Glycine-rich protein GWK isoform 3
Length=34; Score = 43.5 bits (92), Expect = 2e-008
Identities = 19/26 (73%), Positives = 19/26 (73%), Gaps = 2/26 (8%)

```
Query  11  GGGGGLLGGVGGGGG--GAGGGGPGG  34
           GGGGG  GG GGGGG  G GGGG GG
Sbjct  9   GGGGGWKGGGGGGGGWKGGGGGGKGG  34
```

> Yadamp_508|1455|Glycine-rich protein GWK isoform 2
Length=35; Score = 43.5 bits (92), Expect = 2e-008
Identities = 19/26 (73%), Positives = 19/26 (73%), Gaps = 2/26 (8%)

```
Query  11  GGGGGLLGGVGGGGG--GAGGGGPGG  34
           GGGGG  GG GGGGG  G GGGG GG
Sbjct  9   GGGGGWKGGGGGGGGWKGGGGGGKGG  34
```

> CAMPSQ1805; Acanthoscurrin-2; Source: *Acanthoscurria gomesiana* [Tarantula spider], Activity: Antibacterial (Gram-), Antifungal, Target: E. coli SBS363, C. albicans
Length=131; Score = 42.6 bits (90), Expect = 2e-007
Identities = 18/23 (78%), Positives = 18/23 (78%), Gaps = 0/23 (0%)

```
Query  12  GGGGLLGGVGGGGGGAGGGGPGG  34
           GGGGL GG G GGGG GGGG GG
Sbjct  32  GGGGLGGGKGLGGGGLGGGGLGG  54
```

> CAMPSQ1508; Acanthoscurrin-1; Source: *Acanthoscurria gomesiana* [Tarantula spider], Activity: Antibacterial (Gram-), Antifungal, Target: E. coli SBS363, C. albicans
Length=132; Score = 42.6 bits (90), Expect = 2e-007

```
Identities = 18/23 (78%), Positives = 18/23 (78%), Gaps = 0/23 (0%)

Query  12  GGGGLLGGVGGGGGGAGGGGPGG  34
           GGGGL GG G GGGG GGGG GG
Sbjct  32  GGGGLGGGKGLGGGGLGGGGLGG  54
```

Supplementary Information S2 (SI S2):

SI S2. An alignment of amino acid sequences of some identified peptides from protein bands of mucus fraction with MW>20 kDa with known proteins in Gastropoda (taxid:6448), Mollusca (taxid: 6447) and land snails (taxid: 6527) with database Non-redundant protein sequences (nr) and UniProtKB/Swiss-Prot, sequences algorithm blastp (protein-protein BLAST), filtered to match records with percent identity between 50% and 100% (https://blast.ncbi.nlm.nih.gov/Blast.cgi, accessed on 12 April 2024).

TAAFTEDTSVVTGR

> mucus protein [Cornu aspersum] [land snail, non-redundant protein sequences (nr)]

Sequence ID: QEG59314.1, Length: 159, Number of Matches: 1, Score 35.8, bits(77), E value (Expect) 8e-05, Identities 12/14(86%), Positives 12/14(85%), Gaps 0/14(0%)

```
Query  1   TAAFTEDTSVVTGR  14
           TAAFTEDTSV  GR
Sbjct  28  TAAFTEDTSVAEGR  41
```

FTAAFTEDTSVAEGR

> mucus protein [Cornu aspersum] [land snail, non-redundant protein sequences (nr)]

Sequence ID: QEG59314.1, Length: 159, Number of Matches: 1, Score 49.0, bits(108), E value (Expect) 2e-09, Identities 15/15(100%), Positives 15/15(100%), Gaps 0/15(0%)

```
Query  1   FTAAFTEDTSVAEGR  15
           FTAAFTEDTSVAEGR
Sbjct  27  FTAAFTEDTSVAEGR  41
```

GNVAFTAAFTEDTSVAEGR

> mucus protein [Cornu aspersum] [land snail, non-redundant protein sequences (nr)]

Sequence ID: QEG59314.1, Length: 159, Number of Matches: 1, Score 60.4, bits(135), E value (Expect) 4e-13, Identities 19/19(100%), Positives 19/19(100%), Gaps 0/19(0%)

```
Query  1   GNVAFTAAFTEDTSVAEGR  19
           GNVAFTAAFTEDTSVAEGR
Sbjct  23  GNVAFTAAFTEDTSVAEGR  41
```

GALNGNVAFTAAFTEDTSVAEGR

> mucus protein [Cornu aspersum] [gastropoda, non-redundant protein sequences (nr)]

Sequence ID: QEG59314.1, Length: 159, Number of Matches: 1, Score 60.4, bits(135), E value (Expect) 2e-11, Identities 19/19(100%), Positives 19/19(100%), Gaps 0/19(0%)

```
Query  5   GNVAFTAAFTEDTSVAEGR  23
           GNVAFTAAFTEDTSVAEGR
Sbjct  23  GNVAFTAAFTEDTSVAEGR  41
```

GSCNWPMTILMLESLR

RecName: Full=NADH-ubiquinone oxidoreductase chain 6; AltName: Full=NADH dehydrogenase subunit 6 [Albinaria caerulea]

Sequence ID: P48922.1, Length: 155, Number of Matches: 4; Score 22.7 bits(46), E value (Expect) 0.005, Identities 7/9(78%), Positives 7/9(77%), Gaps 1/9(11%)

```
Query  7    MT-ILMLES  14
            MT ILM ES
Sbjct  144  MTSILMVES  152
```

QYLQITWPSPR

> H-type lectin domain-containing protein [Biomphalaria glabrata] [gastropoda, non reduntant protein]

Sequence ID: KAI8776186.1, Length: 683, Number of Matches: 1, Score 31.6, bits(67), E value (Expect) 0.041, Identities 8/8(100%), Positives 8/8(100%), Gaps 0/8(0%)

```
Query  2    YLQITWPS  9
            YLQITWPS
Sbjct  430  YLQITWPS  437
```

VPSDDPGR

> Chain A, Helix aspersa agglutinin (HAA) [Cornu aspersum] [land snail, non-redundant protein sequences (nr)]

Sequence ID: 4Q56_A, Length: 101, Number of Matches: 1, Score 28.2, bits(59), E value (Expect) 0.003, Identities 8/8(100%), Positives 8/8(100%), Gaps 0/8(0%)

```
Query  1   VPSDDPGR  8
           VPSDDPGR
Sbjct  19  VPSDDPGR  26
```

DITFASPYCR

> Chain A, Helix aspersa agglutinin (HAA) [Cornu aspersum] [land snail, non-redundant protein sequences (nr)]

Sequence ID: 4Q56_A, Length: 101, Number of Matches: 1, Score 34.6, bits(74), E value (Expect) 1e-04, Identities 9/10(90%), Positives 10/10(100%), Gaps 0/10(0%)

```
Query  1   DITFASPYCR  10
           +ITFASPYCR
Sbjct  35  NITFASPYCR  44
```

NGGLVHPGPR

> uncharacterized protein LOC112554950 [Pomacea canaliculata] [gastropoda, non-redundant protein sequences (nr)]

Sequence ID: XP_025078819.1, Length: 229, Number of Matches: 1, Score 25.7, bits(53), E value (Expect) 4.5, Identities 8/9(89%), Positives 8/9(88%), Gaps 0/9(0%)

```
Query  2   GGLVHPGPR  10
           GGLV PGPR
Sbjct  30  GGLVYPGPR  38
```

DWTLYVNTPLAPAR

> unnamed protein product, partial [Candidula unifasciata] [land snail, non-redundant protein sequences (nr)]

Sequence ID: CAG5130849.1, Length: 1003, Number of Matches: 2, Score 25.7, bits(53), E value (Expect) 0.33, Identities 7/9(78%), Positives 7/9(77%), Gaps 0/9(0%)

```
Query  2   WTLYVNTPL  10
           W LY NTPL
Sbjct  61  WILYGNTPL  69
```

> mucin 18b [Plakobranchus ocellatus] [gastropoda, non-redundant protein sequences (nr)]

Sequence ID: GFO09821.1, Length: 124, Number of Matches: 7, Score 24.4, bits(50), E value (Expect) 27, Identities 6/8(75%), Positives 6/8(75%), Gaps 0/8(0%)

```
Query  1   DWTLYVNT  8
           DWTLY  T
Sbjct  24  DWTLYLST  31
```

FCPNAQRTR

> glutathione S-transferase omega-1-like [Pomacea canaliculata] [gastropoda, non-redundant protein sequences (nr)]

Sequence ID: XP_025114871.1, Length: 274, Number of Matches: 1, Score 28.6, bits(60), E value (Expect) 0.31, Identities 8/9(89%), Positives 8/9(88%), Gaps 0/9(0%)

```
Query  1   FCPNAQRTR  9
           FCP AQRTR
Sbjct  34  FCPYAQRTR  42
```

> glutathione S-transferase omega-1 [Elysia marginata] [gastropoda, non-redundant protein sequences (nr)]

Sequence ID: GFR70608.1, Length: 410, Number of Matches: 1, Score 28.6, bits(60), E value (Expect) 0.31, Identities 8/9(89%), Positives 8/9(88%), Gaps 0/9(0%)

```
Query  1    FCPNAQRTR  9
            FCP AQRTR

Sbjct  192  FCPYAQRTR  200
```

> Glutathione S-transferase omega-1 [Bulinus truncatus] [gastropoda, non-redundant protein sequences (nr)]

Sequence ID: KAH9494607.1, Length: 287, Number of Matches: 1, Score 28.6, bits(60), E value (Expect) 0.31, Identities 8/9(89%), Positives 8/9(88%), Gaps 0/9(0%)

```
Query  1   FCPNAQRTR  9
           FCP AQRTR
```

```
Sbjct  74  FCPYAQRTR  82
```

NPVGSVPVLELDGK

> glutathione S-transferase omega-1-like [Pomacea canaliculata] [gastropoda, non-redundant protein sequences (nr)]

Sequence ID: XP_025099831.1, Length: 241, Number of Matches: 1, Score 33.7, bits(72), E value (Expect) 0.013, Identities 12/14(86%), Positives 12/14(85%), Gaps 0/14(0%)

```
Query  1   NPVGSVPVLELDGK  14
           NP GSVPVLE DGK
Sbjct  64  NPSGSVPVLEKDGK  77
```

> glutathione S-transferase omega-1-like [Biomphalaria glabrata] [gastropoda, non-redundant protein sequences (nr)]

Sequence ID: KAI8762368.1, Length: 240, Number of Matches: 1, Score 32.9, bits(70), E value (Expect) 0.026, Identities 11/14(79%), Positives 11/14(78%), Gaps 0/14(0%)

```
Query  1   NPVGSVPVLELDGK  14
           NP G VPVLELD K
Sbjct  63  NPNGTVPVLELDNK  76
```

GPCFTPHTYTNWSWLR

> unnamed protein product, partial [Candidula unifasciata] [land snail, non-redundant protein sequences (nr)]

Sequence ID: CAG5121511.1, Length: 301, Number of Matches: 1, Score 34.1, bits(73), E value (Expect) 4e-04, Identities 10/16(63%), Positives 11/16(68%), Gaps 4/16(25%)

```
Query  4   FTPHTY----TNWSWL  15
           FTP+TY    T WSWL
Sbjct  35  FTPQTYKRWMTTWSWL  50
```

DFLPPASLPDFAPSPPRVAER

> unnamed protein product, partial [Candidula unifasciata] [land snail, non-redundant protein sequences (nr)]

Sequence ID: CAG5136685.1, Length: 401, Number of Matches: 1, Score 26.9, bits(56), E value (Expect) 0.34, Identities 10/19(53%), Positives 12/19(63%), Gaps 5/19(26%)

```
Query  1   DFLPPASLPDFAPSPPRVA  19
           ++LP A LPDF     RVA
Sbjct  69  EYLPVAGLPDF-----RVA  82
```

AGYLQITWPSPR

> mucus protein [Cornu aspersum] [land snail, non-redundant protein sequences (nr)]

Sequence ID: QEG59312.1, Length: 340, Number of Matches: 1, Score 40.1, bits(87), E value (Expect) 1e-08, Identities 11/11(100%), Positives 11/11(100%), Gaps 0/11(0%)

```
Query  1    AGYLQITWPSP  11
            AGYLQITWPSP
Sbjct  103  AGYLQITWPSP  113
```

>H-type lectin domain-containing protein [Biomphalaria glabrata]

Sequence ID: KAI8776186.1Length: 683, Number of Matches: 1, Score 31.6 bits(67), E value (Expect) E=0.055, Identities 8/8(100%), Positives 8/8(100%), Gaps 0/8(0%)

```
Query  3    YLQITWPS  10
            YLQITWPS
Sbjct  430  YLQITWPS  437
```

H-type lectin domain-containing protein [Biomphalaria glabrata]

Sequence ID: KAK6961697.1Length: 364Number of Matches: 1 Number of Matches: 1, Score 31.6 bits(67), E value (Expect) E=0.055, Identities 8/8(100%), Positives 8/8(100%), Gaps 0/8(0%)

```
Query  3    YLQITWPS  10
            YLQITWPS
Sbjct  111  YLQITWPS  118
```

VGSNGAR

> mucus protein [Cornu aspersum] [land snail, non-redundant protein sequences (nr)]

Sequence ID: QEG59312.1, Length: 340, Number of Matches: 1, Score 23.1, bits(47), E value (Expect) E=0.004, Identities 7/7(100%), Positives 7/7(100%), Gaps 0/7(0%)

```
Query  1    VGSNGAR  7
```

```
           VGSNGAR
Sbjct  188  VGSNGAR  194
```

ASVTGDLSNK

> mucus protein [Cornu aspersum] [land snail, non-redundant protein sequences (nr)]

Sequence ID: QEG59312.1, Length: 340, Number of Matches: 1, Score 32.0, bits(68), E value (Expect) 8e-04, Identities 10/10(100%), Positives 10/10(100%), Gaps 0/10(0%)

```
Query  1    ASVTGDLSNK  10
            ASVTGDLSNK
Sbjct  93   ASVTGDLSNK  102
```

ELLGNVYRAAFTEDTSVAEGR

> mucus protein [Cornu aspersum] [land snail, non-redundant protein sequences (nr)]

Sequence ID: QEG59314.1, Length: 159, Number of Matches: 1, Score 46.9, bits(103), E value (Expect) 3e-08, Identities 17/22(77%), Positives 18/22(81%), Gaps 1/22(4%)

```
Query  1   ELLGNV-YRAAFTEDTSVAEGR  21
           E  GNV + AAFTEDTSVAEGR
Sbjct  20  EFIGNVAFTAAFTEDTSVAEGR  41
```

ANVTFDLSEK

> von Willebrand factor A domain-containing protein 3B-like isoform X1 [Biomphalaria glabrata] [gastropoda, non-redundant protein sequences (nr)]

Sequence ID: XP_013069929.2, Length: 1663, Number of Matches: 1, Score 28.2, bits(59), E value (Expect) 0.59, Identities 8/8(100%), Positives 8/8(100%), Gaps 0/8(0%)

```
Query  3   VTFDLSEK  10
           VTFDLSEK
Sbjct  23  VTFDLSEK  30
```

> von Willebrand factor A domain-containing protein 3B-like isoform X2 [Biomphalaria glabrata] [gastropoda, non-redundant protein sequences (nr)]

Sequence ID: XP_013069930.2, Length: 1656, Number of Matches: 1, Score 28.2, bits(59), E value (Expect) 0.59, Identities 8/8(100%), Positives 8/8(100%), Gaps 0/8(0%)

```
Query  3   VTFDLSEK  10
           VTFDLSEK
Sbjct  23  VTFDLSEK  30
```

LFSTASGGR

> Collagen alpha-6(VI) chain, partial [Biomphalaria pfeifferi] [gastropoda, non-redundant protein sequences (nr)]

Sequence ID: KAI8767740.1, Length: 799, Number of Matches: 1, Score 26.5 bits(55), E value (Expect) 1.9, Identities 8/9(89%), Positives 9/9(100%), Gaps 0/9(0%)

```
Query  1    LFSTASGGR  9
            +FSTASGGR
Sbjct  708  MFSTASGGR  716
```

> Collagen alpha-6(VI) chain, partial [Biomphalaria pfeifferi]

Sequence ID: KAK0040452.1, Length: 518, Number of Matches: 1, Score 26.5 bits(55), E value (Expect) 1.9, Identities 8/9(89%), Positives 9/9(100%), Gaps 0/9(0%)

```
Query  1    LFSTASGGR  9
            +FSTASGGR
Sbjct  427  MFSTASGGR  435
```

LPLYEDPKLDVSSLR

> uncharacterized protein LOC101853718 [Aplysia californica] [gastropoda, non-redundant protein sequences (nr)]

Sequence ID: XP_012941625.1, Length: 327, Number of Matches: 2, Score 30.3, bits(64), E value (Expect) 0.27, Identities 10/12(83%), Positives 10/12(83%), Gaps 0/12(0%)

```
Query  4    YEDPKLDVSSLR  15
            YE PKLDV SLR
Sbjct  235  YELPKLDVASLR  246
```

FDANPFFSGR

> RecName: Full=Hemocyanin, beta-C chain unit D [Helix pomatia] [land snail, UniProtKB/Swiss-Prot]

Sequence ID: P12031.2, Length: 410, Number of Matches: 2, Score 29.1, bits(61), E value (Expect) 9e-06, Identities 8/9(89%), Positives 8/9 (88%), Gaps 0/9(0%)

```
Query  1    FDANPFFSG  9
            FD NPFFSG
Sbjct  120  FDPNPFFSG  128
```

HGSPIGVPYWDWTR

> hemocyanin alphaN [Cornu aspersum] [gastropoda, non-redundant protein sequences (nr)]

Sequence ID: AYO86684.1, Length: 3432, Number of Matches: 8, Score 52.8, bits(117), E value (Expect) 2e-09, Identities 14/14(100%), Positives 14/14 (100%), Gaps 0/14(0%)

```
Query  1     HGSPIGVPYWDWTR  14
             HGSPIGVPYWDWTR
Sbjct  1784  HGSPIGVPYWDWTR  1797
```

LISEATYFNSR

> hemocyanin beta [Cornu aspersum] [gastropoda, non-redundant protein sequences (nr)]

Sequence ID: AYO86685.1, Length: 3433Number of Matches: 1, Score 38.4 bits(83), E value (Expect) 2e-04, Identities 11/11(100%), Positives 11/11(100%), Gaps 0/11(0%)

```
Query  1     LISEATYFNSR  11
             LISEATYFNSR
Sbjct  1388  LISEATYFNSR  1398
```

> RecName: Full=Hemocyanin, beta-C chain unit D [Helix pomatia] [land snail, UniProtKB/Swiss-Prot]

Sequence ID: P12031.2, Length: 410, Number of Matches: 1, Score 30.3 bits(64), E value (Expect) 6e-05, Identities 9/11(82%), Positives 10/11(90%), Gaps 0/11(0%)

```
Query  1    LISEATYFNSR  11
            LIS ATY+NSR
Sbjct  106  LISKATYYNSR  116
```

FDPNPFFSGR

> RecName: Full=Hemocyanin, beta-C chain unit D [Helix pomatia] [land snail, UniProtKB/Swiss-Prot]

Sequence ID: P12031.2, Length: 410, Number of Matches: 1, Score 33.3, bits(71), E value (Expect) 3e-07, Identities 9/9(100%), Positives 9/9 (100%), Gaps 0/9(0%)

```
Query  1    FDPNPFFSG  9
            FDPNPFFSG
Sbjct  120  FDPNPFFSG  128
```

EVFEQVEHALLAR

Hemocyanin beta [Cornu aspersum] [gastropods, non-redundant protein sequences (nr)]

Sequence ID: AYO86685.1Length: 3433, Number of Matches: 5, Score 34.6 bits(74), E value (Expect) 0.006, Identities 10/10(100%), Positives 10/10(100%), 0/10(0%)

```
Query  4     EQVEHALLAR  13
             EQVEHALLAR
Sbjct  1358  EQVEHALLAR  1367
```

RecName: Full=Hemocyanin, beta-C chain unit D [Helix pomatia] [gastropods, UniProtKB/Swiss-Prot]

Sequence ID: P12031.2, Length: 410, Number of Matches: 3, Score 22.7 bits(46), E value (Expect) 0.050, Identities 7/8(88%), Positives 7/8(87%), Gaps 0/8(0%)

```
Query  4   EQVEHALL  11
           EQVE ALL
Sbjct  76  EQVEEALL  83
```

QYLQITWSPPR

> fibrinogen-like protein A isoform X1 [Biomphalaria glabrata] [gastropoda, non-redundant protein sequences (nr)]

Sequence ID: XP_055864456.1, Length: 383, Number of Matches: 1, Score 24.4, bits(57), E value (Expect) 1.4, Identities 7/9(78%), Positives 7/9 (77%), Gaps 0/9(0%)

```
Query  2    YLQITWSPP  10
            YLQITW  P
Sbjct  105  YLQITWTHP  113
```

TDVTSALLGARCNEGGTNTHSPLR

> unnamed protein product, partial [Candidula unifasciata] [gastropoda, non-redundant protein sequences (nr)]
Sequence ID: CAG5131631.1, Length: 449, Number of Matches: 1, Score 36.7, bits(79), E value (Expect) 0.006, Identities 14/20(70%), Positives 14/20 (70%), Gaps 0/20(0%)

```
Query  1    TDVTSALLGARCNEGGTNTH  20
            TDVT  LLGA    GGTNTH
Sbjct  328  TDVTTSLLGAPYLGGGTNTH  347
```

> unnamed protein product [Candidula unifasciata] [gastropoda, non-redundant protein sequences (nr)]
Sequence ID: CAG5131621.1, Length: 584, Number of Matches: 2, Score 32.0, bits(68), E value (Expect) 0.26, Identities 12/19(63%), Positives 13/19 (68%), Gaps 0/19(0%)

```
Query  2    DVTSALLGARCNEGGTNTH  20
            D+ S L GA   EGGTNTH
Sbjct  462  DIASSLIGAEYGEGGTNTH  480
```

LTQHFNVGSNGAR

> mucus protein [Cornu aspersum] [gastropoda, non-redundant protein sequences (nr)]
Sequence ID: QEG59312.1, Length: 340, Number of Matches: 1, Score 36.7 bits(79), E value (Expect) 0.001, Identities 11/12(92%), Positives 11/12(91%), Gaps 0/12(0%)

```
Query  2    TQHFNVGSNGAR  13
            TQHF VGSNGAR
Sbjct  183  TQHFKVGSNGAR  194
```

>unnamed protein product [Candidula unifasciata]
Sequence ID: CAG5131621.1, Length: 584, Number of Matches: 3, Score 31.2 bits(66), E value (Expect) 0.094, Identities 10/13(77%),Positives 11/13(84%), Gaps 0/13(0%)

```
Query  1    LTQHFNVGSNGAR  13
            LT+HFNVG  GAR
Sbjct  426  LTEHFNVGYKGAR  438
```

>unnamed protein product, partial [Candidula unifasciata]
Sequence ID: CAG5131631.1, Length: 449, Number of Matches: 2, Score 29.5 bits(62), E value (Expect) 0.38, Identities 9/12(75%),Positives 10/12(83%), Gaps 0/12(0%)

```
Query  1    LTQHFNVGSNGA  12
            LT+HF VG NGA
Sbjct  293  LTHHFTVGPNGA  304
```

MPDYDCGCCNGSNGSYGSGGGGGGGR

> unnamed protein product, partial [Candidula unifasciata] [gastropoda, non-redundant protein sequences (nr)]
Sequence ID: CAG5126455.1, Length: 305, Number of Matches: 8, Score 32.5, bits(69), E value (Expect) 0.23, Identities 12/15(80%), Positives 12/15 (80%), Gaps 0/15(0%)

```
Query  11  GSNGSYGSGGGGGGG  25
            G  GSYGS GGGGGG
Sbjct  83  GGGGSYGSSGGGGGG  97
```

> unnamed protein product [Candidula unifasciata] [gastropoda, non-redundant protein sequences (nr)]
Sequence ID: CAG5131834.1, Length: 354, Number of Matches: 8, Score 32.5, bits(69), E value (Expect) 0.24, Identities 15/24(63%), Positives 15/24 (62%), Gaps 9/24(37%)

```
Query  11   GSNGSYG--------SGGGGG-GG  25
            GSNGSYG        SGGGGG GG
Sbjct  254  GSNGSYGGGYGGSAYSGGGGGYGG  277
```

DGMSAAPQAENAFALK

> RecName: Full=Achacin; Flags: Precursor [Lissachatina fulica] [gastropoda, UniProtKB/Swiss-Prot]
Sequence ID: P35903.1, Length: 531, Number of Matches: 3, Score 29.5, bits(62), E value (Expect) 3e-04, Identities 12/17(71%), Positives 13/17 (76%), Gaps 2/17(11%)

```
Query  1    DGMSAAPQA-ENAFALK  16
            DGMSA PQA  +AF LK
Sbjct  251  DGMSALPQALADAF-LK  266
```

> RecName: Full=L-amino-acid oxidase; AltName: Full=Escapin; Flags: Precursor [Aplysia californica] [gastropoda, UniProtKB/Swiss-Prot]
Sequence ID: Q6IWZ0.1, Length: 535, Number of Matches: 1, Score 19.7, bits(39), E value (Expect) 0.95, Identities 5/8(63%), Positives 6/8 (75%), Gaps 0/8(0%)

```
Query  1    DGMSAAPQ  8
            +GMS  PQ
```

```
Sbjct  254  EGMSSVPQ  261
```

SGFPTTSKDR

> RecName: Full=L-amino-acid oxidase; AltName: Full=Escapin; Flags: Precursor [Aplysia californica] [gastropoda, UniProtKB/Swiss-Prot]
Sequence ID: Q6IWZ0.1, Length: 535, Number of Matches: 1, Score 21.4, bits(43), E value (Expect) 0.080, Identities 6/6(100%), Positives 6/6 (100%), Gaps 0/6(0%)

```
Query  4    PTTSKD  9
            PTTSKD
Sbjct  299  PTTSKD  304
```

VGGGPSGVELDEFEARVELK

> RecName: Full=Achacin; Flags: Precursor [Lissachatina fulica] [gastropoda, UniProtKB/Swiss-Prot]
Sequence ID: P35903.1, Length: 531, Number of Matches: 1, Score 21.4, bits(43), E value (Expect) 0.40, Identities 11/27(41%), Positives 11/27 (40%), Gaps 15/27(55%)

```
Query  1   VGGGPSG-------------VELDEFE  14
           VG GPSG             VEL  FE
Sbjct  43  VGAGPSGTYSAYKLRNKGQTVEL--FE  67
```

AELKGDMQYTYADSEKVR

> fibrillin-3, partial [Biomphalaria pfeifferi] [gastropoda, non-redundant protein sequences (nr)]
Sequence ID: KAK0048148.1, Length: 391, Number of Matches: 1, Score 31.2, bits(66), E value (Expect) 0.22, Identities 12/25(48%), Positives 14/25 (56%), Gaps 10/25(40%)

```
Query  4    KGDM--QYTY--------ADSEKVR  18
            KGDM  QY Y        A+SEK+R
Sbjct  157  KGDMNWQYDYWMPIVVGYANSEKIR  181
```

> fibrillin-3, partial [Biomphalaria glabrata] [gastropoda, non-redundant protein sequences (nr)]
Sequence ID: KAK6970092.1, Length: 5172, Number of Matches: 2, Score 31.2, bits(66), E value (Expect) 0.22, Identities 12/25(48%), Positives 14/25 (56%), Gaps 10/25(40%)

```
Query  4     KGDM--QYTY--------ADSEKVR  18
             KGDM  QY Y        A+SEK+R
Sbjct  3269  KGDMSWQYDYWMPIVVGYANSEKIR  3293
```

AKVQVIGVPDDRLLLR

> acyl-CoA synthetase family member 2, mitochondrial-like, partial [Pomacea canaliculata] [gastropoda, non-redundant protein sequences (nr)]
Sequence ID: XP_025086706.1, Length: 514, Number of Matches: 1, Score 35.4, bits(76), E value (Expect) 0.005, Identities 10/11(91%), Positives 11/11 (100%), Gaps 0/11(0%)

```
Query  3    VQVIGVPDDRL  13
            VQVIGVPD+RL
Sbjct  431  VQVIGVPDERL  441
```

> unnamed protein product, partial [Candidula unifasciata] [gastropoda, non-redundant protein sequences (nr)]
Sequence ID: CAG5136482.1, Length: 571, Number of Matches: 1, Score 35.4, bits(76), E value (Expect) 0.005, Identities 10/11(91%), Positives 11/11 (100%), Gaps 0/11(0%)

```
Query  3    VQVIGVPDDRL  13
            VQVIGVPD+RL
Sbjct  491  VQVIGVPDERL  501
```

YVLEDLSAADLELSR

> RecName: Full=FMRFamide-activated amiloride-sensitive sodium channel; AltName: Full=FANACH [Cornu aspersum] [land snail, UniProtKB/Swiss-Prot]
Sequence ID: Q25011.1, Length: 625, Number of Matches: 3, Score 18.5, bits(36), E value (Expect) 0.14, Identities 5/5(100%), Positives 5/5 (100%), Gaps 0/5(0%)

```
Query  3    LEDLS  7
            LEDLS
Sbjct  511  LEDLS  515
```

GSSVSLCVWDWAVLPR

> RecName: Full=FMRFamide-activated amiloride-sensitive sodium channel; AltName: Full=FANACH [Cornu aspersum] [land snail UniProtKB/Swiss-Prot]
Sequence ID: Q25011.1, Length: 625, Number of Matches: 2, Score 15.5, bits(29), E value (Expect) 1.9, Identities 5/7(71%), Positives 5/7 (71%), Gaps 0/7(0%)
```
Query  3    SVSLCVW  9
            SVSL  W
Sbjct  420  SVSLSYW  426
```

MVTSLYPGEDWAMLPR
> mucin-5AC-like, partial [Haliotis rufescens] [gastropoda, non-redundant protein sequences (nr)]
Sequence ID: XP_048239711.1, Length: 740, Number of Matches: 1, Score 31.2, bits(66), E value (Expect) 0.15, Identities 9/16(56%), Positives 11/16 (68%), Gaps 3/16(18%)
```
Query  1   MVTSLYPGEDWAMLPR  16
           M+T LYP +   MLPR
Sbjct  1   MITNLYPAQ---MLPR  13
```

ETVSPGYSEGECTCASITQK
> mucin-5B-like isoform X4 [Haliotis rubra] [gastropoda, non-redundant protein sequences (nr)]
Sequence ID: XP_046552239.1, Length: 790, Number of Matches: 4, Score 27.4, bits(57), E value (Expect) 6.7, Identities 13/21(62%), Positives 14/21 (66%), Gaps 3/21(14%)
```
Query  2    TV--SPGYSEGECTCASITQK  20
            TV  SPG S+G  T A ITQK
Sbjct  261  TVTWSPG-SQGSSTSAPITQK  280
```

HGGGGGGFGGGGFGSR
> uncharacterized protein LOC124113905 [Haliotis rufescens] [molluscs, non-redundant protein sequences (nr)]
Sequence ID: XP_046330350.2, Length: 1175, Number of Matches: 4, Score 38.8, bits(84), E value (Expect) 9e-04, Identities 13/13(100%), Positives 13/13 (100%), Gaps 0/13(0%)
```
Query  2    GGGGGGFGGGGFG  14
            GGGGGGFGGGGFG
Sbjct  322  GGGGGGFGGGGFG  334
```

> fibroin heavy chain isoform X1 [Aplysia californica] [gastropoda, non-redundant protein sequences (nr)]
Sequence ID: XP_012943828.1, Length: 1169, Number of Matches: 28, Score 33.7, bits(72), E value (Expect) 0.018, Identities 13/16(81%), Positives 13/16 (81%), Gaps 3/16(18%)
```
Query  2    GGGGGGFGGG---GFG  14
            GGGGGGFGGG   GFG
Sbjct  608  GGGGGGFGGGPGSGFG  623
```

> elastin-like [Ostrea edulis] [molluscs, non-redundant protein sequences (nr)]
Sequence ID: XP_056017499.1, Length: 757, Number of Matches: 46, Score 34.1, bits(73), E value (Expect) 0.038, Identities 13/15(87%), Positives 13/15 (86%), Gaps 2/15(13%)
```
Query  2    GGGGGGF--GGGGFG  14
            GGGGGGF  GGGGFG
Sbjct  115  GGGGGGFGAGGGGFG  129
```

> RecName: Full=Glycine, alanine and asparagine-rich protein; Flags: Precursor [Haliotis asinina] [molluscs, UniProtKB/Swiss-Prot]
Sequence ID: P86732.1, Length: 507, Number of Matches: 47, Score 26.5, bits(55), E value (Expect) 0.004, Identities 11/17(65%), Positives 11/17 (64%), Gaps 4/17(23%)
```
Query  2    GGGGGGFGGG----GFG  14
            G GG GFGGG    GFG
Sbjct  99   GSGGSGFGGGSGGSGFG  115
```

VFADFELHNIGASADVR
> hemocyanin beta [Cornu aspersum] [gastropoda, non-redundant protein sequences (nr)]
Sequence ID: AYO86685.1, Length: 3433, Number of Matches: 6, Score 56.6, bits(126), E value (Expect) 2e-10, Identities 17/17(100%), Positives 17/17 (100%), Gaps 0/17(0%)
```
Query  1    VFADFELHNIGASADVR  17
            VFADFELHNIGASADVR
Sbjct  327  VFADFELHNIGASADVR  343
```

CQVSLPYWDWAVPLR

> RecName: Full=Hemocyanin, beta-C chain unit G [Helix pomatia] [land snail, UniProtKB/Swiss-Prot]

Sequence ID: P56823.1, Length: 404, Number of Matches: 2, Score 25.2, bits(52), E value (Expect) 5e-04, Identities 5/5(100%), Positives 5/5 (100%), Gaps 0/5(0%)

```
Query  6    PYWDW  10
            PYWDW
Sbjct  98   PYWDW  102
```

LSGYDVSKVNEILK

> hemocyanin beta [Cornu aspersum] [land snail, non-redundant protein sequences (nr)]

Sequence ID: AYO86685.1, Length: 3433, Number of Matches: 3, Score 35.8, bits(77), E value (Expect) 8e-05, Identities 11/13(85%), Positives 11/13 (84%), Gaps 0/13(0%)

```
Query  2     SGYDVSKVNEILK  14
             SGYDV  VNEILK
Sbjct  3216  SGYDVGQVNEILK  3228
```

HLWLLHCFEHDLNGYEYDNLR

> hemocyanin beta [Cornu aspersum] [land snail, non-redundant protein sequences (nr)]

Sequence ID: AYO86685.1, Length: 3433, Number of Matches: 16, Score 40.1, bits(87), E value (Expect) 8e-06, Identities 13/15(87%), Positives 13/15 (86%), Gaps 2/15(13%)

```
Query  8     FEHDLN-GYEYDNLR  21
             FEHD N GYEYDNLR
Sbjct  3201  FEHD-NLGYEYDNLR  3214
```

ANNARLTDASHDNPFSSYTLR

> hemocyanin beta [Cornu aspersum] [land snail, non-redundant protein sequences (nr)]

Sequence ID: AYO86685.1, Length: 3433, Number of Matches: 6, Score 50.7, bits(112), E value (Expect) 1e-09, Identities 15/16(94%), Positives 15/16 (93%), Gaps 0/16(0%)

```
Query  6     LTDASHDNPFSSYTLR  21
             LTDASHDNPFSSYT R
Sbjct  3046  LTDASHDNPFSSYTIR  3061
```

VGRIESESYVVKKSK

> zinc finger protein 502-like [Biomphalaria glabrata] [gastropoda, non-redundant protein sequences (nr)]

Sequence ID: XP_055863608.1, Length: 967, Number of Matches: 1, Score 31.2, bits(66), E value (Expect) 0.12, Identities 10/13(77%), Positives 11/13 (84%), Gaps 0/13(0%)

```
Query  3    RIESESYVVKKSK  15
            RI SES+VVKK K
Sbjct  283  RISSESFVVKKRK  295
```

> zinc finger protein 184 [Biomphalaria glabrata] [gastropoda, non-redundant protein sequences (nr)]

Sequence ID: KAI8795828.1, Length: 967, Number of Matches: 1, Score 31.2, bits(66), E value (Expect) 0.12, Identities 10/13(77%), Positives 11/13 (84%), Gaps 0/13(0%)

```
Query  3    RIESESYVVKKSK  15
            RI SES+VVKK K
Sbjct  283  RISSESFVVKKRK  295
```

VLTFYQSCDCVVDHCHR

> zinc finger protein 664-like [Patella vulgata] [gastropoda, non-redundant protein sequences (nr)]

Sequence ID: XP_050401404.1, Length: 400, Number of Matches: 1, Score 28.6, bits(60), E value (Expect) 1.5, Identities 9/12(75%), Positives 9/12 (75%), Gaps 3/12(25%)

```
Query  1   VLTFYQSCDCVV  12
           VLTFYQ   CVV
Sbjct  6   VLTFYQ---CVV  14
```

GEPGSTGPPGLK

> collagen alpha-1(IV) chain-like, partial [Pomacea canaliculata] [gastropoda, non-redundant protein sequences (nr)]

Sequence ID: XP_025114158.1, Length: 339, Number of Matches: 4, Score 38.4, bits(83), E value (Expect) 2e-04, Identities 12/12(100%), Positives 12/12 (100%), Gaps 0/12(0%)

```
Query  1    GEPGSTGPPGLK  12
            GEPGSTGPPGLK
Sbjct  167  GEPGSTGPPGLK  178
```

NGPSGVELDEFEARVELK

> collagen alpha-1(XIII) chain-like isoform X7 [Haliotis rubra] [gastropoda, non-redundant protein sequences (nr)]

Sequence ID: XP_046573418.1, Length: 327, Number of Matches: 1, Score 28.2, bits(59), E value (Expect) 2.5, Identities 10/17(59%), Positives 12/17 (70%), Gaps 1/17(5%)

```
Query  3    PSGVELDEFEAR-VELK  18
            P G ELDE+  R +ELK
Sbjct  186  PAGLELDEYGQRIIELK  202
```

FVDMDGMFSSAAGGRPEAR

> collagen alpha-4(VI) chain-like isoform X2 [Physella acuta] [gastropoda, non-redundant protein sequences (nr)]

Sequence ID: XP_059154404.1, Length: 273, Number of Matches: 1, Score 35.0, bits(75), E value (Expect) 0.012, Identities 11/14(79%), Positives 12/14 (85%), Gaps 0/14(0%)

```
Query  5    DGMFSSAAGGRPEA  18
            D MFSSA GGRP+A
Sbjct  180  DKMFSSASGGRPDA  193
```

> collagen alpha-1(XII) chain-like [Physella acuta] [gastropoda, non-redundant protein sequences (nr)]

Sequence ID: XP_059163536.1, Length: 692, Number of Matches: 3, Score 31.2, bits(66), E value (Expect) 0.26, Identities 9/12(75%), Positives 10/12 (83%), Gaps 0/12(0%)

```
Query  7    MFSSAAGGRPEA  18
            MF  AAGGRP+A
Sbjct  596  MFGTAAGGRPDA  607
```

RAYTDGMFSSAAGGRPEAR

> collagen alpha-4(VI) chain-like isoform X2 [Physella acuta] [gastropoda, non-redundant protein sequences (nr)]

Sequence ID: XP_059154404.1, Length: 273, Number of Matches: 1, Score 35.0, bits(75), E value (Expect) 0.012, Identities 11/14(79%), Positives 12/14 (85%), Gaps 0/14(0%)

```
Query  5    DGMFSSAAGGRPEA  18
            D MFSSA GGRP+A
Sbjct  180  DKMFSSASGGRPDA  193
```

> collagen alpha-4(VI) chain-like isoform X1 [Physella acuta] [gastropoda, non-redundant protein sequences (nr)]

Sequence ID: XP_059154396.1, Length: 278, Number of Matches: 1, Score 35.0, bits(75), E value (Expect) 0.012, Identities 11/14(79%), Positives 12/14 (85%), Gaps 0/14(0%)

```
Query  5    DGMFSSAAGGRPEA  18
            D MFSSA GGRP+A
Sbjct  185  DKMFSSASGGRPDA  198
```

CNEGGTNTHSPLR

> collagen alpha-6(VI) chain-like [Physella acuta] [gastropoda, non-redundant protein sequences (nr)]

Sequence ID: XP_059164377.1, Length: 1896, Number of Matches: 2, Score 26.1, bits(54), E value (Expect) 6.2, Identities 8/9(89%), Positives 8/9 (88%), Gaps 0/9(0%)

```
Query  4     GGTNTHSPL  12
             GGTNTHS L
Sbjct  1085  GGTNTHSAL  1093
```

NPGYLVSKVNEILK

> mucin-2-like [Physella acuta] [gastropoda, non-redundant protein sequences (nr)]

Sequence ID: XP_059140993.1, Length: 984, Number of Matches: 1, Score 28.6, bits(60), E value (Expect) 0.84, Identities 10/14(71%), Positives 11/14 (78%), Gaps 0/14(0%)

```
Query  1    NPGYLVSKVNEILK  14
            NP  LVSK+ EILK
Sbjct  799  NPTALVSKIKEILK  812
```

> serine/arginine repetitive matrix protein 2 [Biomphalaria glabrata] [gastropoda, non-redundant protein sequences (nr)]
Sequence ID: KAI8735946.1, Length: 1477, Number of Matches: 1, Score 28.6, bits(60), E value (Expect) 0.84, Identities 10/14(71%), Positives 11/14 (78%), Gaps 0/14(0%)

```
Query  1     NPGYLVSKVNEILK  14
             NP  LVSK+ EILK
Sbjct  1307  NPTALVSKIKEILK  1320
```

DCRVRVGGPLFAPSPYLFER
> mucin-17-like [Haliotis rubra] [gastropoda, non-redundant protein sequences (nr)]
Sequence ID: XP_046554219.1, Length: 1822, Number of Matches: 2, Score 29.9, bits(63), E value (Expect) 0.78, Identities 13/25(52%), Positives 14/25 (56%), Gaps 10/25(40%)

```
Query  3     RVRVGGPLF--------APSPYLFE  19
             RVRVG  LF        A SPYLF+
Sbjct  1559  RVRVG--LFDKTKMGGQACSPYLFQ  1581
```

FNFLPLSADALESLR
> E3 ubiquitin-protein ligase HERC2-like isoform X2 [Biomphalaria glabrata] [gastropoda, non-redundant protein sequences (nr)]
Sequence ID: XP_055894969.1, Length: 4995, Number of Matches: 5, Score 29.5, bits(62), E value (Expect) 0.45, Identities 10/12(83%), Positives 10/12 (83%), Gaps 0/12(0%)

```
Query  4     LPLSADALESLR  15
             LPLS DAL SLR
Sbjct  4137  LPLSCDALASLR  4148
```

ASSGSCDFSSSTAANR
> mucin-5AC-like isoform X2 [Haliotis rufescens] [gastropoda, non-redundant protein sequences (nr)]
Sequence ID: XP_048252836.1, Length: 621, Number of Matches: 3, Score 29.9, bits(63), E value (Expect) 0.45, Identities 11/13(85%), Positives 11/13 (84%), Gaps 0/13(0%)

```
Query  2    SSGSCDFSSSTAA  14
            SSGS  FSSSTAA
Sbjct  252  SSGSSSFSSSTAA  264
```

> mucin-5AC-like isoform X1 [Haliotis rufescens] [gastropoda, non-redundant protein sequences (nr)]
Sequence ID: XP_048252834.1, Length: 702, Number of Matches: 3, Score 29.9, bits(63), E value (Expect) 0.45, Identities 11/13(85%), Positives 11/13 (84%), Gaps 0/13(0%)

```
Query  2    SSGSCDFSSSTAA  14
            SSGS  FSSSTAA
Sbjct  333  SSGSSSFSSSTAA  345
```

GCAGCPQAGSK
> fibrillin-1-like [Biomphalaria glabrata] [gastropoda, non-redundant protein sequences (nr)]
Sequence ID: XP_055888733.1, Length: 2868, Number of Matches: 3, Score 26.5, bits(55), E value (Expect) 3.0, Identities 8/9(89%), Positives 8/9 (88%), Gaps 0/9(0%)

```
Query  2    CAGCPQAGS  10
            CAGCPQ GS
Sbjct  960  CAGCPQIGS  968
```
